# Immune Remodeling during Aging and the Clinical Significance of Immunonutrition in Healthy Aging

**DOI:** 10.14336/AD.2023.0923

**Published:** 2024-08-01

**Authors:** Lei Dou, Yang Peng, Bin Zhang, Huiyuan Yang, Kai Zheng

**Affiliations:** ^1^Department of Geriatrics, Tongji Hospital, Tongji Medical college, Huazhong University of Science and Technology, Wuhan 430030, China.; ^2^Department of Surgery, Tongji Hospital, Tongji Medical college, Huazhong University of Science and Technology, Wuhan 430030, China.

**Keywords:** aging, immunonutrition, immunosenescence, inflammaging, gut microbiota, age-related diseases

## Abstract

Aging is associated with changes in the immune system and the gut microbiota. Immunosenescence may lead to a low-grade, sterile chronic inflammation in a multifactorial and dynamic way, which plays a critical role in most age-related diseases. Age-related changes in the gut microbiota also shape the immune and inflammatory responses. Nutrition is a determinant of immune function and of the gut microbiota. Immunonutrion has been regarded as a new strategy for disease prevention and management, including many age-related diseases. However, the understanding of the cause-effect relationship is required to be more certain about the role of immunonutrition in supporting the immune homeostasis and its clinical relevance in elderly individuals. Herein, we review the remarkable quantitative and qualitative changes during aging that contribute to immunosenescence, inflammaging and microbial dysbiosis, and the effects on late-life health conditions. Furthermore, we discuss the clinical significance of immunonutrition in the treatment of age-related diseases by systematically reviewing its modulation of the immune system and the gut microbiota to clarify the effect of immunonutrition-based interventions on the healthy aging.

The world is undergoing a rapid demographic shift toward an aging trend [1]. The quantitative and functional alterations in immune cells, which are a crucial components of aging immunity, and affect both innate and adaptive immunity, are known as immunosenescence [2]. As immunosenescence proceeds, chronic, low-grade, systemic inflammation, named inflammaging, accompanies most if not all age-related diseases [3]. There is sufficient evidence that nutritional intervention has an anti-inflammatory effect in unhealthy aging and age-related diseases [4, 5]. Similarly, the gut microbiota undergoes dramatic changes in composition and function during aging, which may also lead to unhealthy aging and various age-related diseases [6]. Immunonutrients and microecological regulators are increasingly gaining importance in preventing microbiota dysbiosis and aging immune imbalance and reshaping the immune response to confer healthy aging. In this review article, we aimed to detail the characteristics of systemic immunity and the gut microbiota in elderly individuals and age-related diseases related to a dysregulated immune response. We also provide an overview of the roles of immunonutrients and microecological regulators in maintaining aging immune homeostasis, decreasing the development of age-related diseases and in turn promoting healthy aging and longevity.

## Immune features in the elderly

**Figure 1.**
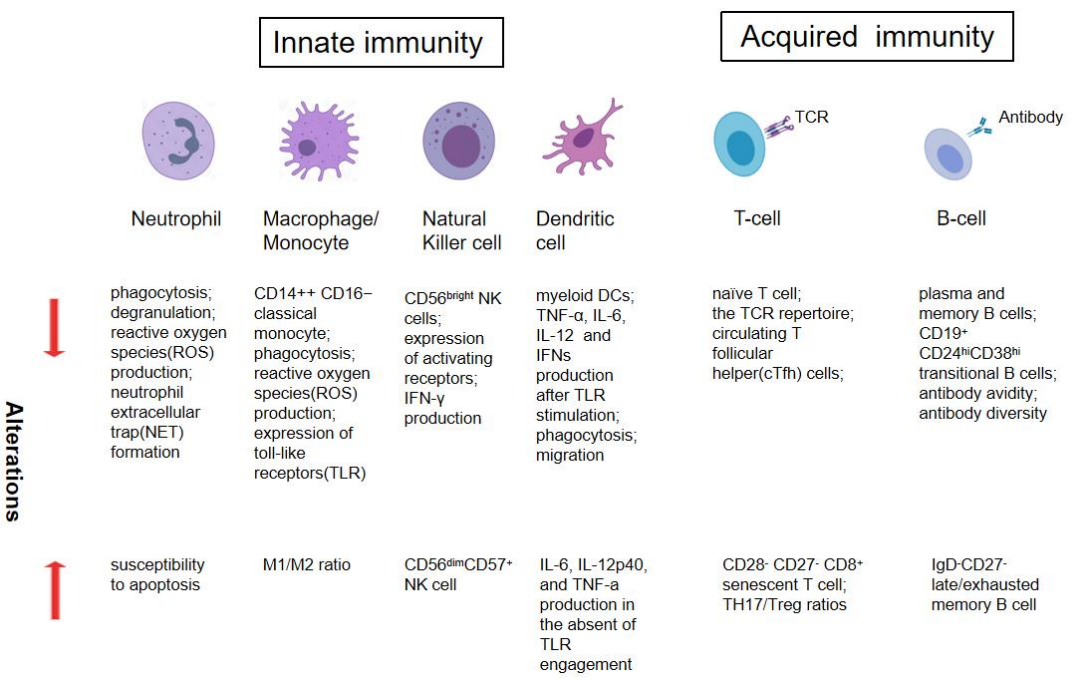
Quantitative and functional alterations in immune cell subpopulations during aging.

As humans age, the immune system suffers a gradual decline in immunity, which manifests as a decreased ability to fight infection, diminished response to vaccination, an increased incidence of cancer, a higher prevalence of autoimmunity and constitutive low-grade inflammation [7]. In addition to cell-intrinsic changes in both innate and adaptive immune cells, as described in Figure 1, alterations in the gut microbiota also play an important role in the age-associated immune dysfunction [8].

### Innate immunity in the elderly

Innate immunity is the host’s first line of defense against invaders. The activation of the innate immune response relies on pattern recognition receptors (PRRs) and creates a bridge to the adaptive immune response [9]. With advanced age, age-related alterations in terms of cell number and function have been described for the cells involved in innate immunity, including dendritic cells (DCs), monocytes, macrophages, mast cells and granulocytes (e.g. neutrophils) [10]. At the same time, their defective migration, phagocytosis and cytokine production abilities can further exacerbate the declines in adaptive immunity, for instance, by not providing efficient antigen presentation to T cells [11]. Finally, dysregulated innate immune signaling leads to chronic, low-grade inflammation, also termed ‘‘inflammaging”, which is thought to contribute to various age-related diseases [3, 12].

With advanced age, alterations in the innate immune cell subpopulations are observed. For example, the numbers of CD14^+^ CD16^-^ classical monocytes and myeloid DCs (mDCs) are reported to be reduced in people aged over 60 years. these cells are derived from myeloid progenitors and have essential roles in phagocytosis, cytokine production, and T-cell priming [13]. However, more generally, hematopoietic stem cells (HSCs) in the bone marrow increase in number with age, which gives rise to increased or stable numbers of innate immune cell subpopulations [14]. For example, circulating neutrophil and monocyte numbers are stable throughout life, and highly differentiated NK cells (CD56^dim^CD57^+^) are reported to increase with age [15].

With advanced age, defective innate immune responses are observed upon stimulation. In elderly individuals, many functions of neutrophils have been reported to decline, including chemotaxis, phagocytosis, ROS production, and neutrophil extracellular trap(NET) release [16]. However, neutrophil susceptibility to apoptosis might be increased due to defective granulocyte-macrophage colony-stimulating factor (GM-CSF) and IL-2 signal transduction [17]. For monocytes and macrophages in advanced age, a decrease in their phagocytic ability contributes to the slow and decreased response to antigen exposure. Additionally, the production of proinflammatory cytokines in response to activation also appears to be impaired, partly due to decreased surface expression of Toll-like receptors (TLRs) [18]. With respect to DCs in elderly individuals, impaired phagocytosis and migration by myeloid DCs (mDCs) negatively impacts their ability to take up antigens [19]. MHC and costimulatory molecule expression following TLR stimulation was found to be impaired in aged mouse models [20]. Furthermore, significantly decreased cytokine production was observed in mDCs following stimulation of TLR1/2, TLR2/6, TLR3, TLR4, TLR5, or TLR8 with multiple different TLR ligands, including IL-6, IL-12p40, and TNF-a [21].

### Adaptive immunity in the elderly

Antigen-specific adaptive immunity has evolved to provide a broader and more finely tuned response against pathogens, allergens and tumors [22]. On the one hand, a diverse population of lymphocytes generates a tremendously diverse repertoire of receptors capable of recognizing components of all potential antigens. On the other hand, a population of long-lived antigen-experienced lymphocytes, named memory lymphocytes, can be quickly activated to generate a more rapid and robust protective response as immunologic memory [23].

With advanced age, altered T- and B-cell subpopulations are observed [24, 25]. For T cells, although there is a shrinking pool of naïve T cells and the TCR repertoire in elderly individuals, the absolute number of T cells remains stable since the number of antigen-experienced memory and effector T cells increases over time. Among CD8+ T cells, a marked decline in the expression of the costimulatory molecules CD27 and CD28 was observed. CD28- CD27- CD8+ T cells have been recognized as a highly differentiated population of T cells, representing the hallmark of immunosenescence in T cells [26, 27]. Among CD4+ T cells, the frequency of circulating T follicular helper (cTfh) cells at baseline is significantly decreased compared to that in young adults [28]. For B cells, the frequencies of both plasma and memory B cells in both the periphery and bone marrow are decreased, which is related to a decline in antibody titers following immunization in elderly individuals [29]. Evidence has demonstrated that the frequency and numbers of immature transitional B cells with the CD19(+) CD24(hi) CD38(hi) phenotype, which is a distinct subset of B cells with immunosuppressive effects, are reduced with age [30].

With advanced age, dysfunction of the adaptive immune response are observed [31]. The expanded CD28- CD27- CD8+ T-cell population shows impaired proliferation ability and IL-2 signaling, which could blunt the ability to generate CD8+ T-cell responses to infection or vaccination [32]. Similarly, Tfh cells from elderly individuals have an impaired ability to promote antigen-specific B-cell proliferation and IgG production [33]. In addition, the increased frequency of regulatory T (Treg) cells in elderly individuals has also been shown to negatively impact the cytotoxic activity of cytotoxic T lymphocytes (CTLs) as well as the production of IL-2 [34]. Regarding the humoral response in elderly individuals, impaired class switching and somatic recombination along with a lower diversity of antibodies are observed compared with those of younger individuals [25, 33]. Due to chronic stimulation, late exhausted memory B cells are expanded and related with increase to an increase in autoantibodies in elderly individuals [35].

### The gut microbiota in the elderly

The human gut has a significant number of bacterial species and other microorganisms, which are collectively referred to as the gut microbiota [36]. The gut wall also represents the largest site of immune tissue, known as gut-associated lymphoid tissue (GALT) [37]. On the one hand, age-related changes in gut physiology modify the microbiota. For example, mucin production decreases with age in mice, which results in a thinner and discontinuous mucus layer and negatively affects the interaction between the microbes and epithelial cells [38]. On the other hand, the relative abundance of some strains of microbiota varies by age, geography and dietary habits [39, 40]. The individual gut microbiota plays a role in the development and function of GALT and the host immune system [41]. With age, there are observable reductions in the size of all lymphoid organs, Ig production, and lymphocyte populations. A decrease in short-chain fatty acid (SCFA)-producing species has also been consistently reported; however, SCFAs are important mediators that promote epithelial cell proliferation and increase the expression of tight junction proteins [42]. Various health-associated behaviors, including diet, lifestyle, and medications, cause older adults to suffer from microbial dysbiosis [43]. Age-related microbial dysbiosis has a close relationship with intestinal permeability, systemic inflammation, and premature mortality [6, 44].

## Age-related immune imbalance and age-related diseases

Age-related remodeling of the immune system plays a major role in many chronic diseases [12]. These age-related chronic diseases are not only the result of aging and inflammaging but may also accelerate the aging process [45]. The gut microbiota also has a central role in inflammaging owing to its ability to release inflammatory products and contribute to crosstalk with other extraenteric organs [46, 47].

### Immunosenescence, immunoaging and age-related diseases

**Figure 2.**
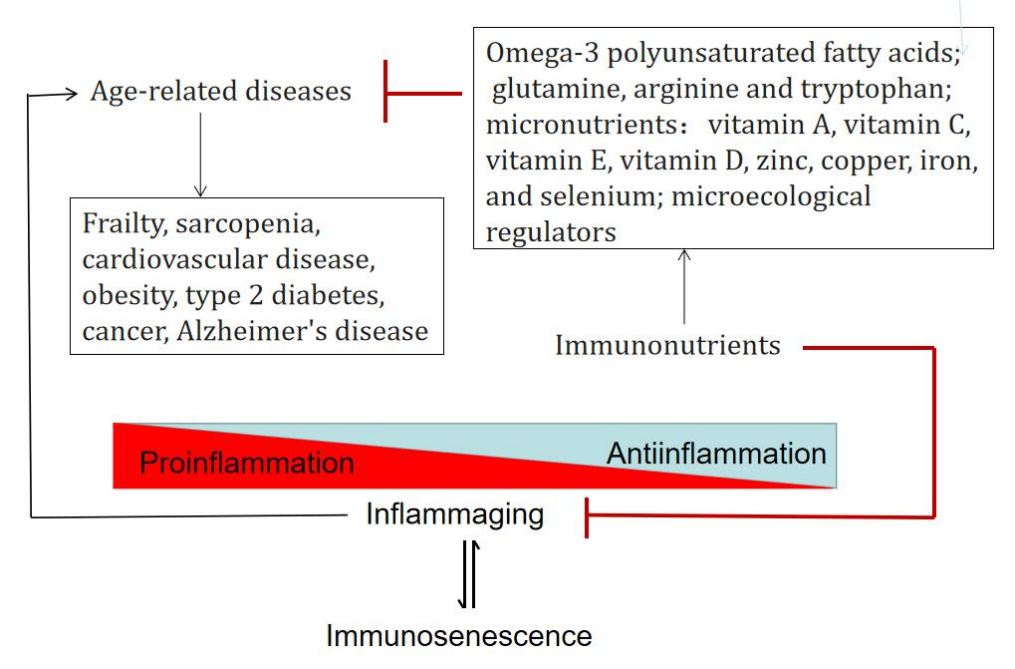
Various immunonutrients alleviate age-related diseases by counteracting the aging immune imbalance from the dual burden of immunosenescence and inflammaging.

During aging, the immune system undergoes remarkable changes, collectively known as immunosenescence, as described above. This immunosenescence is a complex biological phenomenon that affects both natural and acquired immunity [48]. These age-related changes trigger an increase in inflammatory mediators, which together with other modifications, such as an increase in senescent cell numbers, gut microbiota dysbiosis and an unhealthy lifestyle, contribute to inflammaging [3, 49]. When inflammaging cannot induce secondary adaptive activation of anti-inflammatory networks, excessive stimulation of proinflammatory pathways constitutes a driving force and represents the main risk factor for developing age-related diseases and unsuccessful aging. Due to the dual burden of immunosenescence and inflammaging, older adults are more susceptible to infectious diseases and other age-related chronic diseases, as described in Figure 2 [50, 51].

First, it has been demonstrated that older adults experience a greater burden of infectious diseases [e.g., influenza, tuberculosis, pneumonia, and coronavirus disease 2019 (COVID-19)], prolonged infection periods, and increased risk of morbidity and mortality [52]. One important factor is immunosenescence. As aging progresses, immunosenescence can be associated with a reduced ability to mount an effective innate and adaptive immune response to both novel and previously encountered pathogens, including decreased output of immune cells, changes in the ratio of different immune cell subpopulations, and impaired functions of several related immune cells [53]. In addition, inflammaging, a chronic low-grade inflammation, is characterized by elevated serum levels of the acute-phase protein C-reactive protein (CRP) , TNF-α, and IL-6. This elevated inflammatory state reflects sensitized proinflammatory signaling pathways in older people [54]. This state is associated with a lower antibody response to compared to age-matched healthy controls [55].

Next, age has been highly documented as the most important independent risk factor for cancer in nationwide health surveys and epidemiological studies [56]. In terms of cancer occurrence, jeopardizes tumor immune surveillance mechanisms to eradicate malignant cells [57]. At the same time, a persistent chronic inflammatory environment favours genetic instability and subsequent carcinogenesis [58]. A series of clinical observations have also verified the causal link between the cancer occurrence and low-grade chronic inflammation, for example inflammatory bowel disease and colorectal cancer, hepatitis and hepatocellular carcinoma, and smoking-induced lung inflammation and lung cancer. In terms of cancer progression and metastasis, inflammaging is characterized by a shift toward the activation and infiltration of immunosuppressive cells in elderly individuals, including myeloid-derived suppressor cells (MDSCs), tumor associated macrophages (TAMs) and regulatory T cells (Tregs), which are considered essential for premetastatic niche formation and the establishment of a tumor microenvironment(TME) that allows tumor cells to spread [59-61].

In addition, many other age-related diseases may be related to inflammaging either directly or indirectly. Obesity and type 2 diabetes mellitus (T2DM) are related to adipose tissue inflammation. In elderly individuals, the imbalance between pro- and anti-inflammatory adipokines(cytokines secreted specifically by adipocytes) is exacerbated by inflammaging [54]. For atherosclerosis, underlying inflammaging recruits proinflammatory cytokines and immune cells to plaques and leads to worse clinical manifestations of cardiovascular diseases [62]. In Alzheimer disease, immunosenescence and inflammaging may accelerate brain aging, memory loss and neuroinflammation, and result in increased permeability of the blood-brain barrier to proinflammatory mediators and cells from the periphery and the production of amyloid beta (Aβ) peptide [63].

### Gut microbiota dysbiosis and age-related disease

**Figure 3.**
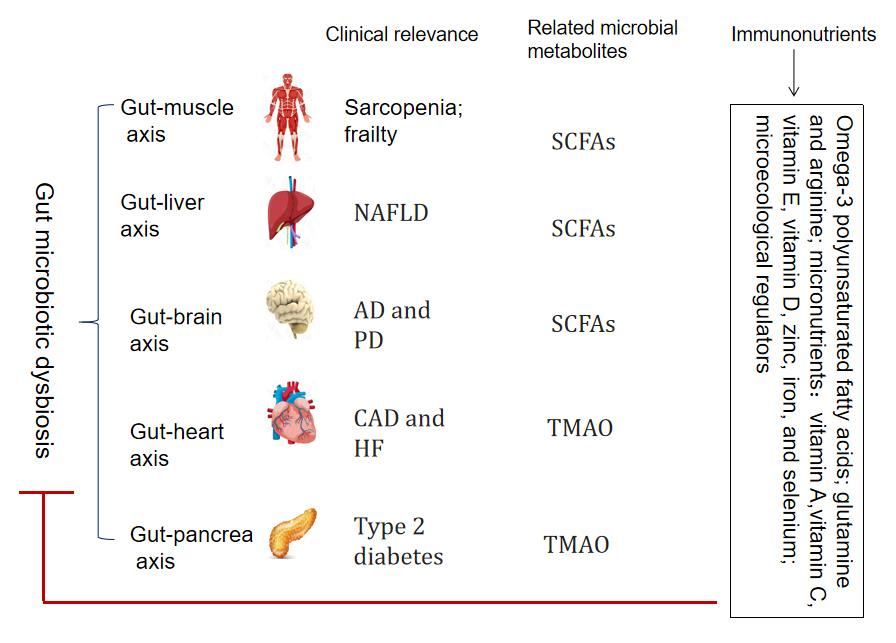
**Various immunonutrients alleviate many chronic diseases associated with gut microbial dysbiosis by affecting the gut-extraenteric tissue axis and related microbial metabolites**. Abbreviations: AD, Alzheimer's disease; CAD, coronary artery disease; HF, heart failure; NAFLD, nonalcoholic fatty liver disease; PD, Parkinson’s disease; SCFAs, short-chain fatty acids; TMAO, trimethylamine-N-oxide.

The microbiota is a key factor in maintaining host homeostasis via functional connectivity with other organs and tissues of the body such as the muscle, bone, liver, heart, brain and pancreas, referred to as the gut-extraenteric tissue axis [64, 65]. Correspondingly, as depicted in Figure 3, age-related changes in the gut microbiota appear to influence the onset and progression of enteric and extraenteric diseases, including colorectal cancer, sarcopenia and physical frailty, nonalcoholic fatty liver disease(NAFLD), coronary heart disease, neurodegenerative diseases, and type 2 diabetes mellitus [46, 66].

Alterations in the gut microbiota in aged individuals are associated with increased incidences of enteric diseases. Several individual bacterial species have been associated with colorectal cancer, including Strepto-coccus bovis (S. bovis), Bacteroides fragilis (ETBF), Enterococcus faecalis (E. faecalis), and Escherichia coli (E. coli) [67]. Bacterial stimulation of immune responses can cause continuous low-grade inflammation, resulting in tumorigenesis. Due to gut inflammatory conditions in elderly individuals, the abundances of SCFA-producing bacteria such as Bifidobacterium, Faecalibacterium, and Blautia were reduced and the integrity of the intestinal barrier was impaired, which is also associated with colorectal cancer risk [68].

Immunosenescence and Inflammaging are crucial mechanisms of gut-muscle axis aging, which is characterized by loss of muscle mass and reduced muscle function [69, 70]. The decreased relative abundances of Faecalibacterium and Bifidobacterium in the aged gut microbiota are negatively correlated with muscle strength. Microbiota-derived SCFAs can influence skeletal muscle cell function by promoting mitochondrial activity [71]. However, the age-related proinflammatory gut micro-environment exhibits reduced gut levels of SCFAs [72]. These findings indicated that gut microbiota dysbiosis was actively involved in the pathogenesis of sarcopenia and physical frailty, and could increase the risk of negative outcomes such as falls, fractures, disability and mortality [73].

In elderly patients, gut microbiota dysbiosis and related gut mucosal barrier dysfunction are closely correlated with NAFLD development, which implies gut-liver axis impairment [74]. The relative abundances of Bacteroides and Ruminococcus were significantly increased in patients over 60 years of age with biopsyproven NAFLD. The severity of NAFLD was associated with a shift in the metabolic function of the gut microbiota such as carbohydrate, lipid, and amino acid metabolism [75]. For example, Bacteroides is negatively correlated with the levels of fecal SCFAs and amino acids, and decreased contents of SCFAs and branched-chain fatty acids might be detrimental for NAFLD [76]. In addition, increased gut permeability may facilitate the passage of lipopolysaccharide (LPS) and other inflammatory factors to the blood, decrease the availability of choline and increase ethanol production in the intestine [77]. In the end, these pathologic changes affect NAFLD in different ways.

Bidirectional interactions between the gut and brain are associated with age-related neurodegenerative diseases including Alzheimer’s disease (AD) and Parkinson’s disease (PD) [78]. Gut microbiota dysbiosis-associated intestinal permeability-induced inflammation has been shown to increase the risk of AD [79]. The decreased Bacteroides abundance in AD patients was significantly correlated with cognitive impairment and brain amyloidosis. Gut dysbiosis can also stimulate the differentiation and proliferation of brain-infiltrated Th1 immune cells, contributing to AD-associated neuroinflammation by MI microglia activation [80]. In PD, increased Firmicutes and decreased Prevotella abundances were shown to be associated with disease progression. Gut microbiota dysbiosis can promote α-synuclein (αSyn)-mediated motor deficits and brain pathology. Insoluble aggregates and oligomeric forms of αSyn may further activate microglia [81]. Bacterial metabolites such as SCFAs can also modulate microglial homeostasis and promote their full maturation [82]. However, the production of SCFAs is affected by microbiota dysbiosis.

A decreased abundance of gut microbes with the capacity to produce butyrate and elevated circulating levels of trimethylamine-N-oxide (TMAO) have been linked with cardiovascular diseases, such as coronary artery disease (CAD) and heart failure (HF) [83]. For example, reduced relative abundances of Eubacterium rectale and Dorea longicatena from the Lachnospiraceae family and lower levels of Faecalibacterium from the Ruminococcaceae family were found in older patients with HF [84]. Depletion of SCFAs contributes to the pathogenesis and prognosis of heart failure with reduced ejection fraction (HFpEF) by affecting numerous factors in the body, such as obesity, hypertension, myocardial hypertrophy and fibrosis [85]. These findings indicated that SCFA-producing bacteria in the gut microbiota may be potential targets to prevent the progression of HFpEF.

Type 2 diabetes mellitus (T2DM) is another typical disease associated with gut microbial dysbiosis. Ruminococcus, Collinsella, and Bifidobacteriaceae are enriched in the gut microbiota from elderly subjects with T2DM [86]. Various microbial metabolic alterations, such as TMAO levels, are associated with low-grade inflammation that plays an important role in T2DM development, including effects on carbohydrate metabolism, starch and sucrose metabolism, phenylpropanoid biosynthesis, and the biosynthesis of amino acids [87]. The mechanism by which intermittent fasting alleviates the diverse stages of T2DM has been shown to be related to gut microbiota modification [88].

The gut microbiota is also involved in the regulation of bone metabolism at different levels [89]. Actinomyces and Clostridium species were more prevalent in subjects with osteoporosis. Gut microbial dysbiosis impairs the intestinal absorption of calcium and dysregulates osteoclast activity via serum levels of insulin-like growth factor 1 (IGF-1) [90]. Moreover, microbiota alterations also reduce bone strength and quality and affect the OPG/RANKL pathway in osteoclasts [91].

## Host metabolites and immunonutrient interventions for immune improvement in the elderly

It is well known that adequate nutrition is an important factor allowing for the normal development of the immune system as well as its function throughout life, especially in elderly adults who are more vulnerable to malnutrition [92]. Metabolite-mediated inflammation, termed metaflammation, is recognized as a major source of inflammaging that develops with the aging process [12]. Fortified immunonutrient complements are considered a safe and cost-effective means for older people to improve their immune status, although aging itself fundamentally alters the impact of nutrition on immune function [93, 94]. As shown in Figure 2 and Figure 3, various immunonutrients have been shown to alleviate age-related diseases and to counteract unhealthy aging related to gut micriobiota dysbiosis by maintaining aging immune homeostasis and a healthy gut microbiota.

### Lipid-related immunonutrients for aging immune system homeostasis and clinical relevance

The process of aging is accompanied by chronic immune activation. Omega-3 polyunsaturated fatty acids (PUFAs), such as eicosapentaenoic acid (EPA) and docosahexaenoic acid (DHA), are the most studied fatty acids that have significant anti-inflammatory properties [95]. The level of intake of long-chain PUFAs was inversely associated with proinflammatory markers (IL-6, IL-1, and TNF-α) and was positively associated with anti-inflammatory markers (IL-10 and TGFβ) in an elderly population [96]. As an immunonutrient, modulation of immune and inflammatory responses in the elderly has been reported with an increased intake of PUFAs of the n-3 series, although high-quality observational and intervention studies are lacking. N-3 PUFA supplementation for 3 months in a cohort of patients with congestive heart failure was associated with a significant decrease in serum IL-6 and TNF-α levels [97]. Supplementation with n-3 PUFA in patients with AD showed beneficial effects on the inhibition of cognitive decline associated with neuroinflammation [98]. In patients with sarcopenia, there is also a positive effect of n-3 PUFA supplementation on overall body muscle mass and strength [99]. In the setting of coronary artery disease and chronic obstructive pulmonary disease, a weak benefit of n-3 PUFAs was related to clinical outcomes by limiting and resolving inflammatory processes [100].

Accumulating evidence has revealed that n-3 PUFAs modulate host immunity by acting on gut microbes [101]. High levels of n-3 PUFAs in fish oil exert health benefits on the gut microbiota by increasing the abundance of beneficial bacteria, such as Bifidobacteria, and subsequently inhibiting the inflammatory response associated with metabolic endotoxemia [102]. An imbalanced intake of n-3/n-6 PUFAs may lead to an increase in the Firmicutes-to-Bacteroidetes ratio (F/B ratio), which is associated with diabetes, nonalcoholic fatty liver disease (NAFLD), and other age-related diseases [103, 104].

### Amino acid-related immunonutrients for aging immune system homeostasis and clinical relevance

Amino acids, such as glutamine, arginine and tryptophan, have been linked to the modulation of immune responses [105]. However, aging is associated with dysregulated amino acid metabolism. Indeed, amino acid metabolism dysregulation is associated with the development of several age-related diseases, including type 2 diabetes, cardiovascular disease, Alzheimer's disease and frailty [106, 107].

Glutamine is a conditionally essential amino acid in catabolic states during stress that is utilized by immune cells and possesses immunomodulatory properties [108]. However, advanced age is accompanied by a decline in glutamine levels in endothelial cells and reduced cellular synthesis [109]. Glutamine constitutes an essential factor for lymphocyte proliferation and cytokine production, macrophage phagocytic activities and bacterial killing by neutrophils [110]. Supplementation with glutamine could have a beneficial role in age-associated functional defects. For example, glutamine supplementation enhances the strength and power of knee muscles and improves glycemic control and plasma redox balance in exercising elderly women [111]. Glutamine is also effective in inducing repolarization of macrophages from an M1 to an M2 phenotype to prevent obesity- or diabetes-associated pathologies [112].

Glutamine also plays an important role in gut health and the crosstalk between gut microbes and the immune system via different mechanisms including reducing the F/B ratio, reducing bacterial translocation, and increasing the production of secretory immunoglobulin A (SIgA) and immunoglobulin A+ (IgA+) cells in the intestinal lumen [113]. Glutamine supplementation results in a diminished incidence of infection in critically ill patients or patients after surgery, including elderly patients [114]. Glutamine supplementation can improve intestinal function by increasing the abundance of intestinal-friendly microbiota from the phyla Bacteroidetes and Actinobacteria and can thereby ameliorate constipation [113]. Patients with intestinal mucosal damage such as stomatitis and mucositis, which may be induced by chemotherapy and radiotherapy, may also benefit from oral glutamine supplementation [115].

Arginine is a conditionally essential amino acid that has been identified as the nitrogen source for nitric oxide generated by endothelial and immune cells in vasodilatory and host-defense mechanisms and is involved in regulating the normal function of the cardiovascular and immune systems [116]. As an immunonutrient, arginine supplementation has been proposed to maintain normal immune function. Arginine metabolites, such as methylated arginine asymmetric dimethylarginine (ADMA) and the arginine analog homoarginine, are related to risk stratification in many key chronic diseases in old age, including cardiovascular disease, chronic obstructive pulmonary disease, dementia, and depression [117]. Immunosuppressive cells suppress the function of effector immune cells by catabolizing L-arginine through the activation of arginase 1 (ARG1), which is associated with a compensatory anti-inflammatory response during aging and is often overexpressed in age-related diseases [118]. The roles of protein arginine methyltransferases (PRMTs) in regulating immune cells, cancer cells, and cardiovascular and neuronal function have also been investigated and are linked to many chronic diseases [119]. Recently, in a nontuberculous mycobacterial pulmonary disease (NTM-PD) mouse model, oral administration of L-arginine resulted in enrichment of the gut microbiota composition with Bifidobacterium species and increased protective host defense in the lungs against NTM-PD [120].

Tryptophan is an essential amino acid found in many protein-based foods. The levels of plasma tryptophan are determined by a balance between dietary intake and its removal from plasma, which plays a pivotal role in immune system regulation [121]. One of the key enzymes that degrades free tryptophan in the kynurenine pathway is indoleamine-2,3-dioxygenase (IDO). IDO can be induced by various inflammatory stimuli, such as Th1-type cytokine interferon-gamma stimulation [122]. Enhanced tryptophan breakdown rates in elderly individuals have been linked with several age-related diseases with increased proinflammatory immune activation [123]. An increased kynurenine/tryptophan ratio can be detected in the activated immune system in older adults, which reflects IDO activity [124]. Chronic low-grade inflammation during aging can also lead to an elevation in circulating kynurenine levels. The activation of the tryptophan-degrading enzyme IDO can be an integral defense mechanism to prevent overwhelming immune reactions and to induce an immunotolerant status [125]. Changes in the microbiota composition are connected to tryptophan consumption [126]. A potential increase in tryptophan consumption by the gut microbiota may affect tryptophan bioavailability to the host. There is a relationship between reduced serum tryptophan levels and increased immune activation [127]. In an accelerated aging Ercc1^-/Δ7^ mouse model, dietary tryptophan restriction can increase gut microbial diversity by arresting B-cell development, which is associated with a reduction in the abundance of Akkermansia spp. in the gut microbiota [128].

### Micronutrients as immunonutrients for aging immune system homeostasis and clinical relevance

A higher prevalence rate of inadequate micronutrient intake in elderly people has been reported both in the community and in long-term care facilities, where it impacts various functions within the immune system, manifesting as reduced resistance to infections and an increase in the severity of symptoms [129]. Micronutrient supplementation, including vitamin A, vitamin C, vitamin E, vitamin D, zinc, copper, iron, and selenium, to restore concentrations to recommended levels is necessary to maintain normal immune function, particularly after an infection or in older people with damaged immunity [130]. For example, higher levels of CD4+ and CD8+ T cells and an increased lymphocyte proliferative response to mitogens have been reported with vitamin A, C and E supplementation [130]. Supplementation with multiple micronutrients in older people also results in higher postvaccination immune responses, increased lymphocyte responses to mitogens, and enhanced NK cell activity [131]. In addition, zinc deficiency can profoundly change immune system homeostasis, which involves both innate and adaptive immunity, causing damaged phagocytosis and intracellular killing activity of phagocytes, reduced NK-cell activity and T-cell proliferation [132]. However, 1 month of daily supplementation with 440 mg of zinc sulfate significantly elevated the proportion of circulating T cells, DTH response, and antitetanus toxin IgG titers in healthy elderly individuals [133]. A sufficient zinc supply could also prevent degenerative age-associated diseases, including infection and cancer [134].

The bioaccessibility and bioavailability of micronutrients are related to the gut microbiome, and the consumption of micronutrients also affects the composition of the gut microbiota [135]. For example, studies have shown that the gut microbiome can be modulated by increasing the abundance of presumed commensals (by supplementing with vitamins A, D, and E), increasing or maintaining microbial diversity (vitamins A and C) and richness (vitamin D), increasing short-chain fatty acid production (vitamin C), or increasing the abundance of short-chain fatty acid producers (vitamin E) [136]. Consumption of Fe supplements reduces the amount of lactic acid bacteria (bifidobacteria and lactobacilli) and increases enteropathogenic Escherichia coli, which is associated with intestinal inflammation [137]. Zn deficiency in mice can negatively alter the microbiota composition and gut-brain signaling and can then trigger an increase in inflammatory markers [138].

### Microecological regulators as “immunonutrients” for gut microbiome homeostasis and clinical relevance

Microecological regulators, including probiotics, prebiotics, synbiotics, and postbiotics, are increasingly gaining importance in preventing and/or reverting age-related imbalances in the gut microbiota and conferring healthy antiaging benefits [139, 140]. Microecological regulators have been found to modify the population of the gut microflora and have been shown to modulate both local and systemic immunity. Microecological regulator intake may affect downstream immunoregulatory pathways and improve recovery from gastrointestinal disorders, such as antibiotic-associated diarrhea, inflammatory bowel disease and colorectal cancer, and other diseases at distant sites, such as upper respiratory tract infections and AD [141]. Several clinical studies have examined the effects of microecological regulator supplementation in elderly people. For example, the administration of fermented milk containing Lactobacillus johnsonii La1 may contribute to suppressing infections by improving nutritional and immunological homeostasis [142]. Pooled data from eligible trials showed that probiotics significantly increased NK cell activity in healthy elderly individuals [143]. Probiotic supplementation might affect gut microbiota dynamics and influence cognitive function in older adult populations with Alzheimer’s disease, mild cognitive impairment or healthy conditions [79, 144]. Supplementation with probiotic strains(such as L. fermentum, L. plantarum, B. coagulans, and B. subtilis) reduces the production of circulating proinflammatory cytokines (e.g., TNF-α, IL-6, and IFN-γ) and/or increases the production of anti-inflammatory cytokines such as IL-10 [145, 146]. Overall, microecological regulator supplementation can modify the gut microbiota and further benefit systemic immune function by improving the gut microbial balance. Improvements in age-related immune defects and clinical outcomes of age-related disease have been demonstrated, depending on the dose and bacterial strains.

## Conclusions and perspectives

During aging, dramatic quantitative and qualitative changes have been revealed in terms of the systemic and gut immune systems. Dysregulated aging immunity represents a major risk factor for most age-related diseases in terms of an imbalance among inflammaging and immunosenescence and gut microbial dysbiosis. Various immunonutrients have been shown to maintain aging immune homeostasis and a healthy gut microbiota, and to then promote healthy and successful aging, including omega-3 polyunsaturated fatty acids, glutamine, arginine, tryptophan, micronutrients and microecological regulators.

There are inconsistent data about the effect of immunonutrition on clinical outcomes in the elderly population, especially in some settings. For example, in the DO-HEALTH randomized clinical trial, treatment with vitamin D3 (2000 IU/d) and omega-3 fatty acids (1 g/d) did not result in statistically significant differences in the improvement in systolic or diastolic blood pressure, nonvertebral fractures, physical performance, infection rates, or cognitive function among adults aged 70 years or older without major comorbidities [147]. Some studies suggest that Fe supplementation is potentially harmful to the gut microbiota [148]. Therefore, specific immunonutrient supplementation should be performed with caution to avoid potential adverse effects. In addition, when considering the impact of single or multiple immunonutrients on immunosenescence, inflammaging, and the gut microbiota, it is important to recognize that background diet is an important variable because the imbalanced consumption of these nutrients can result in adverse impacts on metabolic processes as well as on immune function and inflammatory processes [149].

There is no doubt that the idea of an immunonutrition-based intervention to maintain aging immune homeostasis and alleviate age-related diseases is appealing, although the level of evidence has sometimes been considered to be low. Indeed, many other confounding variables should be considered to properly identify the effects of specific target immunonutrients in elderly populations, including the timing and duration of supplementation, baseline nutritional status, intake of other nutrients, medication use and clinical state. Therefore, more large-scale, long-term, well-designed studies are required to properly identify the effects of specific immunonutrients in target patient populations. Furthermore, the molecular mechanism by which immunonutrients and microbial metabolites regulate the aging immune system should be focused on in the future to provide insight into the beneficial effects of immunonutrition-based interventions to counteract unhealthy aging and to treat age-related diseases.

In future studies, researchers should decrease the effect of confounding factors in the experimental design. For example, many older people suffer from multiple disease coexisting states. When we discuss the results of immunonutrition intervention, detailed subgroup analyses should be performed because the baseline clinical state is a significant confounding factor. Areas for future research should also address the role of immunonutrient supplementation in vaccination efficacy. Hence, strategies should be developed to ensure optimal nutrition for older adults to increase their health-span and reduce health care costs associated with their care. Further scientific evidence on immune metabolism will help to further clarify the regulatory role of immunonutrition in the aging immune system and its clinical significance.
